# Supplementary material for: Diagnostic Performance and Clinical Utility of Conventional PCR Assay in Early Diagnosis of COVID-19 Associated Rhino-Orbito-Cerebral Mucormycosis
Source: J Fungi (Basel). 2022 Aug 11;8(8):844. doi: 10.3390/jof8080844 (PMC9409716; doi:10.3390/jof8080844)
Supplement: Supplementary file 1 [file jof-08-00844-s001.zip › jof-1797552-supplementary.pdf]

SUPPLEMENTARY TABLES:

Table S1 : Baseline characteristics of patients (n=33)

Characteristics	No of Patients (%)	Characteristics	No of Patients (%)
**1) Gender**		Home Isolation	17 (51.52 %)
Male	24 (72.73%)	Oxygen Therapy	12 (36.36%)
Female	09 (27.27%)	Corticosteroid Therapy	15 (45.45%)
**2) Age Groups (in years)**		Ventilator Support	04 (12.12%)
<20 years	0 (00.00%)	**7) Co-morbidities**	
20-40 years	05 (15.15%)	Preexisting Diabetes	19 (57.57%)
41-60 years	23 (69.70%)	Diabetes developed during COVID treatment	03 (09.09%)
> 60 years	05 (15.15%)	Hypertension	05 (15.15%)
**3) VA at Presentation**		CHD with L-R shunt	01 (03.03%)
20/20-20/40	18 (54.55%)	Bronchitis with COPD	02 (06.06%)
20-50-20/200	07 (21.21%)	**8) Duration between onset of symptoms and presentation**	
<20/200 – No PL	08 (24.24%)	1-7 days	12 (36.36%)
**4) Laterality**		8-14 days	08 (24.24%)
Right Eye	17 (51.51%)	Beyond 14 days	13 (
Left Eye	16 (48.48%)	**9) Duration between COVID infection and onset of ROCM symptoms**	
**5) Initial Presenting Hospital**		1- 7 days	06 (18.18%)
General Hospital	14 (42.42%)	8-14 days	12 (36.36%)
Tertiary Eye Care Centre	19 (57.58%)	15-21 days	09 (27.27%)
**6) COVID 19 Infection related Risk factors**		Beyond 21 days	06 (48.48%)
Hospitalization	16 (48.48%)		

Table S2: Clinical manifestations (N=33)

Clinical Findings	No of Patients (%)	Imaging and Endoscopy Findings	No of Patients (%)
**Ocular / Orbital**			
Decreased Vision	09 (27.27%)	Orbital Bony erosion	06(18.18%)
Ptosis	08 (24.24%)	EOM Thickening	11 (33.33%)
Ophthalmoplegia	17 (51.52%)	Medial Orbit involvement	18 (54.55)
Ocular and Periorbital Pain	22 (66.67%)	Lateral Orbit Involvement	01 (03.03%)
Proptosis	09 (27.27%)	Superior + Medial + Inferior Orbit Involvement	01(03.03%)
Lid edema	14 (42.42%)	Sub-periosteal abscess	01(03.03%)
Lid abscess	01(03.03%)	Cavernous sinus and Orbital Apex	09 (27.27%)
Autoevisceration of globe	01 (03.03%)	NA	-
Conjunctiva congestion	13 (3939%)	NA	-
**Facial**			
Facial Edema	16(48.48%)	Pterygopalatine fossa Involvement	01(03.03%)
Facial Numbness & Paresthesia	06 (18.18%)	NA	-
Skin Eschar	01 (03.03%)	NA	-
**Oral**			
Palatal Ulcer	03 (09.09%)	NA	-
Gingival edema with Loosening of Teeth	05 (15.15%)	NA	-
**Nasal**			
Nasal Discharge	12 (36.36%)	Normal Sinuses	03 (09.09%)
Nasal Bleed	07 (21.21%)	Isolated Ethmoid Sinusitis	03 (09.09%)
Eschar on nasal cavity	14 (42.42%)	Isolated Maxillary Sinusitis	05 (15.15%)
NA	-	Ethmoid + Maxillary Sinusitis	11 (33.33%)
NA	-	Ethmoid + Maxillary + Frontal	04 (12.12%)
NA	-	Ethmoid + Shenoid +Maxillary	03 (09.09%)
NA	-	Pansinusitis (Ethmoid + Maxillary + Frontal + Sphenoid)	04 (12.12%)

Table S3: Diagnostic performance of PCR assay and nucleotide sequencing

Patient Sl No	Lab Id	Sample type	Culture report	Identification of Fungus after LPCB	PCR Results	Identification of fungus after sequencing	Accession number assigned
1	570/21	Nasal biopsy	S/o Mucor	Rhizopus sp.	Mucor	R. oryzae	MZ412886
2	579/21	Nasal biopsy	S/o Mucor	Rhizopus sp.	Mucor	R. oryzae	
	579/21	Orbital mass	S/o Mucor	Rhizopus sp.	Mucor	R. oryzae	MZ412887
3	584/21	Nasal biopsy	No growth	NA	Negative	Not done	NA
	584/21	Orbital mass	Fungus	Aspergillus niger	Fungus	A. niger	MZ412888
	584/21	Endonasal swab	No growth	NA	Fungus	A. niger	NA
4	585/21	Nasal biopsy	S/o Mucor	Rhizopus sp.	Mucor	R. oryzae	MZ412889
	585/21	Endonasal swab	No growth	NA	Mucor	R. oryzae	NA
5	592/21	Nasal biopsy	Fungus	Unidentified hyaline fungus	Mucor & Fungus	Not done	
	592/21	Orbital mass	Fungus	Unidentified hyaline fungus	Fungus	Schizophyllum commune	MZ540883
	592/21	Endonasal swab	No growth	NA	Mucor	R. oryzae	MZ412890
6	595/21	Nasal biopsy	Fungus	Asperigilus flavus	Mucor & Fungus	R. oryzae and A. flavus	MZ416884 R. oryzae MZ434748 A. flavus
	595/21	Endonasal swab	No growth	NA	Negative for both	Not done	
7	598/21	Nasal biopsy	S/o Mucor	Rhizopus sp.	Mucor & Fungus	R. oryzae	MZ412892
	598/21	Endonasal swab	Fungus	Asperigilus flavus	Negative for both	Not done	
8	607/21	Nasal biopsy	No growth	NA	Fungus	Penicillium sclerotiorum	MZ434750
	607/21	Endonasal swab	No growth	NA	Negative for both	Not done	
9	616/21	Nasal biopsy	S/o Mucor and A. Flavus	Rhizopus sp.&A. flavus	Mucor & Fungus	R. delemar & A. flavus	MZ434749 A. flavus MZ412891 R. delemar
	616/21	Endonasal swab	No growth	ND	Mucor & Fungus	Not done	
	616/21	Orbital mass	No growth	NA	Mucor & Fungus	Malassezia restricta	MZ648416 M.restricta
10	632/21	Nasal biopsy	S/o Mucor	Rhizopus sp.	Mucor & Fungus	R. oryzae	MZ412893
	632/21	Endonasal swab	No growth	NA	Mucor & Fungus	Malassezia restricta	MZ648415
11	636/21	Nasal biopsy	Fungus (Candida sp.)	Candida albicans	Mucor & Fungus	R. delemar	MZ412895

	636/21	Endonasal swab	No growth	NA	Mucor & Fungus	Candida glaebosa	MZ416886
12	637/21	Nasal biopsy	No growth	NA	Mucor & Fungus	R. oryzae	MZ412896
	637/21	Endonasal swab	No growth	NA	Mucor & Fungus	Not identified (Faint band)	
13	654/21	Nasal biopsy	No growth	NA	Mucor & Fungus	Not identified (Faint band)	
	654/21	Endonasal swab	No growth	NA	Mucor & Fungus	Not identified (Faint band)	
	654/21	Control	No growth	NA	Negative for both	Not identified (Faint band)	
14	655/21	Nasal biopsy	S/o Mucor	Rhizopus sp.	Mucor & Fungus	R. oryzae	MZ416887
	655/21	Endonasal swab	No growth	NA	Fungus	Not identified due to faint band	
15	681/21	Nasal biopsy	No growth	NA	Mucor & Fungus	R. oryzae	MZ434751
	681/22	Endonasal swab	No growth	NA	Mucor & Fungus	Malassezia restricta	MZ540428
16	685/21	Nasal biopsy	Fungus	Unidentified hyaline fungus	Mucor & Fungus	Not identified due to faint bands	
	685/22	Endonasal swab	Fungus	Asperigilus niger	Mucor & Fungus		
	685/23	Orbital mass	No growth	Not done	Mucor	Not identified due to faint bands.	
17	691/21	Nasal biopsy	S/o Mucor	Rhizopus sp.	Mucor & Fungus	Rhizopus oryzae	MZ648417
	691/22	Endonasal swab	No growth		Mucor & Fungus	Rhizopus oryzae	
	691/21	Orbital mass	S/o Mucor	Rhizopus sp.	Fungus	Citeromyces matritensis	MZ684136
	691/24	Brain tissue	No growth	NA	Mucor	Rhizopus oryzae	
18	733/21	Nasal biopsy	No growth	NA	Mucor & Fungus	Citeromyces matritensis	MZ540430
	733/22	Endonasal swab	No growth	NA	Mucor & Fungus		
19	738/21	Nasal biopsy	No growth	NA	Mucor & Fungus	Not identified due to faint bands.	
	738/22	Endonasal swab	No growth	NA	Fungus	Citeromyces matritensis	MZ540431
20	762/21	Nasal biopsy	No growth	NA	Fungus	Citeromyces matritensis	MZ648419
21	791/21	Nasal biopsy	No growth	NA	Fungus	Candida glaebosa	MZ684138
	791/22	Endonasal swab	No growth	NA	Mucor	R. microsporous	MZ684139
22	795/21	Nasal biopsy	No growth	NA	Fungus	Schizophyllum commune	MZ684140
	795/22	Endonasal swab	No growth	NA	Negative for both	NA	
23	796/21	Nasal biopsy	No growth	NA	Negative for both	NA	
24	798/21	Nasal biopsy	No growth	NA	Fungus	Candida glaebosa	MZ701974
	798/22	Endonasal swab	No growth	NA	Negative for both	Not done	

25	833/21	Nasal Biopsy	No growth	NA	Mucor & Fungus	Not identified due to faint bands.	
	833/22	Endonasal Swab	No growth	NA	Mucor & Fungus	Not identified due to faint bands.	
26	834/21	Nasal Biopsy	No growth	NA	Mucor & Fungus	Not identified due to faint bands.	
	834/22	Endonasal Swab	Fungus	UIH	Fungus	Coprinopsis cinerea	MZ701975
27	835/21	Endonasal Swab	No growth	NA	Mucor & Fungus	Not identified due to faint bands.	
28	1135/21	Endonasal Swab	No growth	NA	Fungus	Malassezia restricta	MZ416888
29	995/21	Nasal Biopsy	Fungus	Exserohilum Species	Fungus	Not identified due to faint bands.	
		Endonasal Swab	No growth	NA	Fungus	Not identified due to faint bands.	
30	1077/21	Nasal Biopsy	No growth	NA	Mucor & Fungus	Not identified due to faint bands.	
		Endonasal Swab	No growth	NA	Negative for both	NA	
31	590/21	Nasal biopsy	No growth	NA	Negative for both	NA	
32	1145/21	Nasal Biopsy	Fungus (Candida sp.)	Candida sp.	Fungus	Candida duobushaemulon ii	OK523373
		Endonasal Swab	No growth		Fungus	NA	
	1195/21	Lid abscess	Mucor and Fungus	Rhizopus sp. & Asp. Flavus	Mucor and Fungus	Not identified due to faint bands.	
33	1118/21	Orbital mass	Fungus	UIH	Fungus	Nectaromyces rattus	OK523372
